# Latamoxef for Neonates With Early-Onset Neonatal Sepsis: A Study Protocol for a Randomized Controlled Trial

**DOI:** 10.3389/fphar.2021.635517

**Published:** 2021-06-09

**Authors:** Hui Qi, Yue-E Wu, Ya-Li Liu, Chen Kou, Ze-Ming Wang, Xiao-Xia Peng, Liang Chen, Hong Cui, Ya-Juan Wang, Jie-Qiong Li, Wei Zhao, A-Dong Shen

**Affiliations:** ^1^ Beijing Key Laboratory of Pediatric Respiratory Infection Diseases, Key Laboratory of Major Diseases in Children, Ministry of Education, National Clinical Research Center for Respiratory Diseases, National Key Discipline of Pediatrics (Capital Medical University), Beijing Pediatric Research Institute, Beijing Children’s Hospital, Capital Medical University, National Center for Children’s Health, Beijing, China; ^2^ Department of Clinical Pharmacy, Key Laboratory of Chemical Biology (Ministry of Education), School of Pharmaceutical Sciences, Cheeloo College of Medicine, Shandong University, Jinan, China; ^3^ Center for Clinical Epidemiology and Evidence-based Medicine, Beijing Children’s Hospital, Capital Medical University, National Center for Children’s Health, Beijing, China; ^4^ Department of Neonatology, Beijing Obstetrics and Gynecology Hospital, Capital Medical University, Beijing, China; ^5^ Department of Pediatrics, Beijing Friendship Hospital, Capital Medical University, Beijing, China; ^6^ Department of Neonatology, Children’s Hospital, Capital Institute of Pediatrics, Beijing, China; ^7^ Department of Clinical Pharmacy, Clinical Trial Center, The First Affiliated Hospital of Shandong First Medical University and Shandong Provincial Qianfoshan Hospital, Shandong Engineering and Technology Research Center for Pediatric Drug Development, Shandong Medicine and Health Key Laboratory of Clinical Pharmacy, Jinan, China; ^8^ Children’s Hospital Affiliated to Zhengzhou University, Henan Children’s Hospital, Zhengzhou Children’s Hospital, Beijing, China

**Keywords:** latamoxef, early-onset neonatal sepsis, neonate, randomized controlled trial, study protocol

## Abstract

Early-onset neonatal sepsis (EONS), a bacterial infection that occurs within 72 h after birth, is associated with high likelihood of neonatal mortality. Latamoxef, a semi-synthetic oxacephem antibiotic developed in 1980s, has been brought back into empirical EONS treatment in recent years. In the preliminary work, we established a population pharmacokinetics (PPK) model for latamoxef in Chinese neonates. Moreover, in order to better guide clinical treatment, we conducted dose simulation and found that ascending administration frequency could improve the target rate of 70% of patients having a free antimicrobial drug concentration exceeding the MIC during 70% of the dosing interval (70% fT > MIC). Accordingly, this study is aimed to compare the 70% fT > MIC, efficacy and safety between conventional regimen and PPK model regimen for rational use of latamoxef in EONS treatment. A single-blind, multicenter randomized controlled trial (RCT) for latamoxef will be conducted in Chinese EONS patients. Neonates (≤3 days of age, expected number = 114) admitted to the hospital with the diagnosis of EONS and fulfilling inclusion and exclusion criteria will be randomized (ratio of 1:1) to either a conventional regimen (30 mg/kg q12h) or model regimen (20 mg/kg q8h) latamoxef treatment group for at least 3 days. Primary outcome measure will be 70% fT > MIC and secondary outcome indicators will be the latamoxef treatment failure, duration of antibiotic therapy, changes of white blood cell count (WBC), C-reactive protein (CRP) and procalcitonin (PCT), blood culture results during administration and incidence of adverse event (AE)s. Assessments will be made at baseline, initial stage of latamoxef treatment (18–72 h) and before the end of latamoxef treatment. Ethical approval of our clinical trial has been granted by the ethics committee of the Beijing Children’s Hospital (ID: 2020-13-1). Written informed consent will be obtained from the parents of the participants. This trial is registered in the Chinese Clinical Trial Registry (ChiCTR 2000040064).It is hoped that our study will provide a clinical basis for the rational clinical use of latamoxef in EONS treatment.

## Introduction

Neonatal sepsis, a leading cause of mortality in neonates worldwide, is divided into early-onset (EONS) and late-onset neonatal sepsis (LONS) (Polin, 2012; Shane et al., 2017). EONS occurs within 72 h after birth and the mortality rate of EONS was reported as high as 30% in high-income countries and up to 60% in low-income countries (Thaver and Zaidi, 2009; Stoll et al., 2011). Accordingly, rational anti-infective treatment of EONS plays a crucial role in preventing neonatal mortality and protecting the health of neonates. However, antibiotic resistance, off-label use and adverse reactions plague EONS treatment. Because the prevalence of antibiotic-resistant bacterial strains has increased dramatically, recently there has been a renewed interest in historical antibiotics for EONS treatment (Thaver and Zaidi, 2009; Stoll et al., 2011), such as latamoxef. Latamoxef, a second-generation semi-synthetic oxacephem antibiotic, developed in 1980s, is mainly used to treat infections, caused by Gram-positive and -negative aerobic, as well as anaerobic bacteria (Carmine et al., 1983). Although latamoxef has been used for the anti-infective treatment of neonates since 1980s, limited neonatal pharmacokinetics (PK) data and off-label use remain a vexing problem for this drug being used in the field of EONS treatment.

To assess the PK features of latamoxef in neonates, we performed a population pharmacokinetics (PPK) study of latamoxef in Chinese neonates and established a PPK model for them (Carmine et al., 1983). Current body weight, birth weight, and postnatal age have been identified as significant covariates influencing latamoxef clearance (Qi et al., 2019). Moreover, to develop a rational dosing regimen for latamoxef, we conducted dose simulation and found that ascending administration frequency could improve the target rate of 70% of patients having a free antimicrobial drug concentration exceeding the MIC during 70% of the dosing interval (70% fT > MIC) (Qi et al., 2019). Based these findings, to provide more pharmacodynamics (PD) data for generalizing this PPK model-based regimen, we plan to conduct a single-blind, multicenter randomized controlled trial (RCT) and compare the 70%fT > MIC, efficacy and safety between conventional regimen and PPK model regimen for latamoxef in EONS treatment for the first time.

## Methods and Design

### Trial Design

**FIGURE 1:**
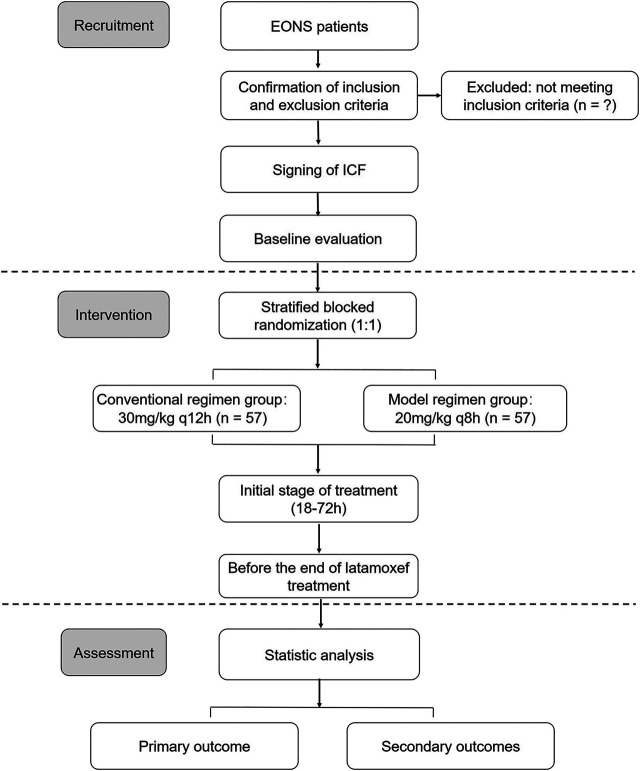
The schematic diagram of the trial design.

The proposed clinical trial will be a randomized, single-blind and multicenter intervention study for latamoxef in EONS treatment. This trial is registered in the Chinese Clinical Trial Registry (ChiCTR 2000040064) and is in full adherence to the principles of the Declaration of Helsinki and Good Clinical Practice (GCP) guidelines. This trial will be conducted at three hospitals in China (Table 1), and 114 EONS patients will be randomly enrolled to two regimen groups with a ratio of 1:1 from November 2020 to December 2021. The schematic diagram of study design is shown in Figure 1.

**TABLE 1:** Hospitals participating in this RCT.

Code	Participating hospitals
1	Beijing friendship hospital
2	Beijing obstetrics and gynecology hospital
3	Children’s hospital, Capital institute of pediatrics

### Ethics and Dissemination

Our research group will protect the rights and safety of participants by full compliance with the Declaration of Helsinki and Good Clinical Practice (GCP) guidelines. Ethical approval of this trial has been granted by the ethics committee of the Beijing Children’s Hospital (ID: 2020-13-1). Written informed consent will be obtained from the parents of the participants. Adequate measures will be taken to ensure confidentiality of data collected in this trial. Results of this trial will be disseminated to the public through relevant academic and professional journals, academic conferences and workshops.

### Diagnostic Criteria of Early-Onset Neonatal Sepsis

The diagnostic criteria of EONS will refer to expert Consensus on the Diagnosis and Management of Neonatal sepsis (version 2019) (The Subspecialty Group of Neonatology, 2019).

1) The suspected diagnosis of postnatal age in days (PNA) ≤3 contains any of the following: abnormal clinical manifestations, the mother has chorioamnionitis and premature rupture of membranes (PROM) ≥18 h. Sepsis can be ruled out if there are no abnormal clinical manifestations, including negative blood culture, and less than two consecutive non-specific blood tests (white blood cell count, immature neutrophil count, platelet count, C-reactive protein (CRP) and procalcitonin (PCT), etc.) at 24 h intervals.
2) The patient has been clinically diagnosed with clinical abnormalities (Table 2) and met any of the following conditions at the same time: the blood non-specific tests ≥2 positive, the cerebrospinal fluid (CSF) test suppurative meningitis change, the pathogenic DNA detected in the blood.
3) When the patient has clinical manifestations (Table 2), blood culture or CSF (or other sterile cavity fluid) culture positive, and the diagnosis confirmed.

**TABLE 2:** Clinical manifestations of neonatal sepsis.

System	Clinical manifestations
Whole body	Fever, hypothermia, poor response, poor feeding, edema, low apgar score
Digestive system	Jaundice, abdominal distension, vomiting or gastric retention, diarrhea and hepatosplenomegaly
Respiratory system	Dyspnea, apnea, cyanosisetc.
Circulatory system	Pale face, cold limbs, bradycardia, tachycardia, marbled skin, hypotension or capillary filling time >3 s
Urinary system	Oliguria and renal failure
Blood system	Bleeding and purpura

### Inclusion Criteria

Patients will be recruited for this study if they meet all of the following criteria:

1) PNA ≤ 3;
2) Term infants and preterm infants with gestational age (GA) ≥ 32 weeks;
3) The EONS diagnostic criteria;
4) Suitable for latamoxef treatment;
5) Written informed consent signed by the parent or legal guardian of the neonates.

### Exclusion Criteria

Patients with any of the following exclusion criteria shall not be admitted to this study:

1) Expected survival time shorter than the duration of the treatment cycle;
2) Severe congenital malformations;
3) Having the high risk of serious bleeding, such as disseminated intravascalar coagulation (DIC) and Vitamin K deficiency bleeding (VKDB) (Rajagopal et al., 2017; Araki and Shirahata, 2020):
  A. Decreased platelet count (≤150 × 10^9^/L);
  B. Significantly prolonged prothrombin time (PT, especially PT is more than twice over upper limit of the normal range);
  C. Prolonged activated partial thromboplastin time (APTT, is more than the upper limit of the normal range);
  D. INR ≥4 or a value >4 times the normal values in the presence of normal platelet count and fibrinogen level;
4) Undergoing surgery within the first week of birth;
5) Receiving other trial drug treatment;
6) Having other factors that researchers believe are not suitable for inclusion.

### Recruitment Strategies

Inpatient neonates will be enrolled from Beijing Friendship Hospital, Beijing Obstetrics and Gynecology Hospital, and Children’s Hospital, Capital Institute of Pediatrics (Table 1). We’ll use advertisements for recruitment on social media, such as WeChat, QQ, etc. Associate chief physicians in the neonatal units of these three hospitals will be in charge of EONS patient recruitment.

### Randomization and Blinding

To minimize selective bias, Beijing Six Yuan Space Information Technology Co., Ltd (Six Yuan) has been entrusted to conduct a stratified blocked randomization by using a computer random number generator in the system of Six Yuan. Participants will be randomly divided into the conventional treatment group and the model treatment group with a ratio of 1:1. The randomized code of the trial, derived from the random system of Six Yuan, will be the unique identification code of participants. After assignment of the randomization code, researchers are un-blinded to the treatment regimen of latamoxef. Considering that the drug concentration detection and data analysis will significantly affect the conclusion of this trial, all the research assistants in charge of drug concentration detection and statisticians will be blinded to treatment assignment until the trial is completed.

### Intervention

After inclusion in the study, participants will be randomized into a conventional treatment group and a model treatment group. They will receive the following interventions at least 3 days. Conventional treatment group: latamoxef, 30 mg/kg q12h; model treatment group: latamoxef, 20 mg/kg, q8h. As steady-state plasma concentration of latamoxef will be reached after 18 h of dosing, the opportunistic blood sampling method will be used to collect samples for testing plasma drug concentration at initial stage of the treatment (18–72 h) (Table 3).

**TABLE 3:** Study schedule of latamoxef RCT in EONS treatment.

Items	Study phases (The beginning of latamoxef treatment is set as 0 h)		
	Baseline (−3–−1 day)	Initial stage of treatment (18–72 h)	Before the end of latamoxef treatment
Informed consent form (ICF)	X		
Demographic information	X		
Medical history	X		
Diagnosis and determination of the treatment regimen	X		
Inclusion/exclusion criteria	X		
Get the random number	X		
Comorbid drug use	X	X	X
Weight	X	X	X
Vital signs^a^ and physical examination	X	X	X
Blood routine^b^	X	X	X
Blood culture	X	X	X
CRP and PCT	X	X	X
Liver and kidney function^c^ ^d^	X	X	X
Cerebrospinal fluid (CSF) examination (optional)^e^	X	X	X
Dose, frequency and time point of latamoxef administration		X	X
Detection of plasma latamoxef concentration		Random sampling point	
Sampling time of plasma samples		X	
Efficacy assessment			X
Adverse event assessment		X	X
Case report form (CRF)	X	X	X

a Vital signs include the temperature, blood pressure and oxygen saturation (SaO_2_).

b Blood routine tests include red blood cells, hemoglobin, white blood cells, and platelets.

c Liver function tests include alanine and aspartate aminotransferases, alkaline phosphatase level, total bilirubin, and gamma-glutamyl transferase.

d Kidney function tests include creatinine and blood urea nitrogen.

e CSF examination include CSF routine (characters, cells and Pandy’s test) and culture. This examination can be used when neonatologists suspect a patient has a neurological infection.

### Outcome Measures

#### Primary Outcome

The primary outcome will be 70% fT > MIC, which is the target rate of 70% of patients having a free antimicrobial drug concentration exceeding the MIC during 70% of the dosing interval. 70% fT > MIC is appropriate to evaluate therapeutic efficacy of time dependent antibiotics in neonates (Craig, 1995; Wu et al., 2020). Based on PPK model of latamoxef, 70% fT > MIC will be calculated after testing plasma drug concentration at the initial stage of treatment (Table 3).

#### Secondary Outcomes

This trial has five secondary outcomes:

1) Rate of latamoxef treatment failure: the symptoms and laboratory indicators of infection persist or worsen after latamoxef treatment. Neonatologists have to increase of the dose of latamoxef, or add other antibiotics to the treatment with latamoxef, or stop latamoxef treatment and switch to other antibiotics.
2) Duration of antibiotic therapy: the length of antibiotic therapy for EONS;
3) Changes of white blood cell count (WBC), CRP and PCT: comparison of the changes of WBC, CRP and PCT at baseline, initial stage of treatment (18–72 h) and before the end of treatment (Table 3);
4) Blood culture results during administration: comparison of blood culture results at baseline, initial stage of treatment (18–72 h) and before the end of treatment (Table 3);
5) Incidence of adverse events (AE)s: monitoring and recording AEs during latamoxef therapy (Table 3).

### Safety and AE Monitoring

In this trial, safety will be monitored, including AEs, serious AEs, and withdrawals due to AEs. The safety indexes are mainly composed of the routine blood, liver function, kidney function, coagulation function and vital signs. AEs of latamoxef mainly include anaphylaxis (such as rashes, urticaria, and itching), gastrointestinal reactions (such as vomiting, diarrhea and abdominal pain), and other latamoxef associated AE (such as anaphylactic shock, elevated levels of aminotransferases and serious bleeding).

### Sample Size Estimates

According to our preliminary PPK study and dose simulation for latamoxef in neonates (Qi et al., 2019), 60.2% of neonates using conventional treatment regimen (30 mg/kg q12 h) reached 70% fT > MIC and 80.1% of neonates using model treatment regimen (20 mg/kg q8h) reached 70% fT > MIC with MIC of 2 mg/L, respectively. We conducted sample size estimation for superiority design by using PASS 15.0 (NCSS, Kaysville, Utah, United States). To achieve a statistical power of 80% (one-sided type 1 error of 5%), the calculated sample size of each treatment group was 57 patients per treatment group (114 in total) with the ratio of 1:1. Taking into account a 10% dropout rate, 64 patients per treatment group (128 in total) will be required.

### Study Data and Statistical Analysis Plan

The procedures and contents of data collection for this trial are detailed in Table 2. Demographic information, diagnosis, clinical data and laboratory data at baseline will be recorded on electronic case report form (eCRF) platform provided by Six Yuan (Table 2). In addition, clinical data, laboratory data and AEs at every planed time point after initiation of treatment will also be recorded in this eCRF platform (Table 2). According to the data collection methods and standards formulated by project director and researchers, all data of eCRF platform will be recorded in a true, detailed and careful manner to ensure the authenticity of the data.

Statisticians, independent of all the other processes, will conduct the statistical analyses by using SPSS version 15.0 for Windows (Chicago, IL, United States). The statisticians will calculate the mean, standard deviation, median, minimum, maximum, lower quartile (Q1), upper quartile (Q3) for quantitative data and describe numbers and percentages for qualitative data (Wang et al., 2020). Comparisons between two groups will be conducted as following: t-tests or Wilcoxon rank-sum for normal or nonnormal ability distributions for quantitative data; chi-square test or Fisher’s exact test for qualitative data (Wang et al., 2020). Analysis for missing data performed on the intention-to-treat principle. For all analysis, *p* value < 0.05 will be considered statistically significant.

### Quality Control

In order to ensure the quality of the trial, project director and researchers from each hospital have formulated detailed project implementation protocol and emergency plan at the beginning of this trial. During the clinical trial, researchers participating in this experiment shall undergo unified training to clarify and unify the recording methods and standards for data collection on the eCRF platform. Moreover, the clinical inspector, designated by the director, will make an inspection tour to each hospital periodically to ensure that the researchers strictly adhere to the clinical trial protocol and fill in the information correctly.

## Discussion

In our proposed study, based on our preliminary PPK-PD analysis of latamoxef in neonates, we are aimed to conduct a single-blind, multicenter RCT and compare the 70%fT > MIC, efficacy and safety between conventional regimen and PPK model regimen for latamoxef in EONS treatment for the first time. Our study will provide PD data for optimizing latamoxef usage in EONS treatment.

EONS, a serious threat for health of neonates, is mainly caused by bacterial pathogens transmitted vertically from mother to infant before or during delivery (Hornik et al., 2012). The organisms involved in EONS are not identical in different countries and regions. In the developed countries, *Group B Streptococcus* (*GBS*) and *Escherichia coli* (*E. coli*) are most frequently bacteria; in China, *E. coli* and *Coagulase-negative staphylococcus* (*CONS*) were the leading pathogenic bacteria (Jiang et al., 2019), followed by *Achromobacter xylosoxidans* (*A. xylosoxidans*) and *Klebsiella pneumoniae* (*K. pneumoniae*). Therefore, broad-spectrum cephalosporins are often used as an important anti-infective drug in the Chinese EONS treatment, such as latamoxef. However, due to the limited PK and PD neonatal data, the dosing guidelines and optimal treatment regimen of latamoxef cannot be applied in EONS treatment.

To ensure that the finding of this trial can comprehensively reflect PD characteristics of latamoxef in EONS treatment, 70% fT > MIC will be used as the primary outcome indicator, and efficacy and safety indexes will be employed as the secondary outcome indicators. Because there’s no clinical evaluation of latamoxef in EONS treatment, the trial design is derived from other clinical trials about cephalosporin treatment or therapeutic decision conducted in children or neonates (Stocker et al., 2017; Wu et al., 2020). Rate of treatment failure, duration of antibiotic therapy, changes of inflammatory indicators, results of blood culture and incidence of AEs are commonly used indicators in efficacy and safety evaluation for anti-infective agents (Molyneux et al., 2011; Stocker et al., 2017). Since latamoxef is an oxacephem antibiotic that imparts time-dependent bactericidal effects, fT > MIC as a PD parameter is essential for efficacy evaluation of latamoxef. Although 40–50% fT > MIC is usually used in adult antimicrobial treatment, a goal of 70% fT > MIC is considered as a more conservative endpoint for avoiding treatment failure in immunologically immature neonates (Craig, 1995; Craig, 2001).

According to the bacterial susceptibility data of latamoxef in relation to the bacteria commonly observed in Chinese EONS patients, the MIC90 values of *E. coli* and *K. pneumoniae* were 1 mg/L and 2 mg/L (Cui Lan-Qing, 2016). Thus, 70% fT > MIC targets in two treatment regimens with MIC of 2 mg/L were applied as parameters in sample size calculation.

Our study had some limitations. First, since EONS patients often improve or recover at hospital discharge, follow-up is not designed in this trial. Hence our efficacy evaluation may miss some relapse situation after hospital discharge. Second, due to the few positive culture results and antimicrobial susceptibility results of bacteria in neonatal clinical practice, we chose 70% fT > MIC target with MIC of 2 mg/L in sample size calculation based on epidemiological microbiology data instead of individual microbiology. Finally, our study data are only from Chinese EONS patients in Beijing, limiting the generalizability to other populations and areas with different epidemiological microbiology data.

We hoped that our study can provide reliable data to support rational use of latamoxef in EONS treatment.
